# The prognostic value of EGFR overexpression and amplification in Esophageal squamous cell Carcinoma

**DOI:** 10.1186/s12885-015-1393-8

**Published:** 2015-05-08

**Authors:** Dongxian Jiang, Xiaojing Li, Haixing Wang, Yuan Shi, Chen Xu, Shaohua Lu, Jie Huang, Yifan Xu, Haiying Zeng, Jieakesu Su, Yingyong Hou, Lijie Tan

**Affiliations:** 1 Department of Pathology, Zhongshan Hospital, Fudan University, Shanghai, 200032 People’s Republic of China; 2 Department of Thorax Surgery, Zhongshan Hospital, Fudan University, Shanghai, 200032 People’s Republic of China

**Keywords:** Esophageal squamous cell carcinoma, Epidermal growth factor receptor, Immunohistochemistry scoring system, Fluorescence in situ hybridization

## Abstract

**Background:**

In view of the prominent role in cancer cell biology and alteration in substantial numbers of ESCC, defining EGFR molecular characteristics relevant to patient prognosis is of great importance. Therefore, we analyzed the protein expression and gene copy variation of the epithelial growth factor receptor (EGFR) in Chinese esophageal squamous cell carcinoma (ESCC) and explored the possible associations with various features of the tumors and survival of the patients.

**Methods:**

Sections were made from tissue microarray composed of 96 ESCC, and examined for EGFR expression by means of immunohistochemistry (IHC) and for EGFR gene amplification by means of fluorescence in situ hybridization (FISH). The results of IHC were evaluated with six different reported scoring systems. Correlation with clinical features and survival was evaluated using chi-square test and Kaplan–Meier analysis.

**Results:**

EGFR overexpression according to scoring system 1 significantly correlated with advanced lymph node involvement (*P* = 0.046), patient disease specific free survival (DFS) (*P* = 0.006) and overall survival (OS) (*P* = 0.007). No such association was observed using other 5 scoring systems (*P* > 0.05 ). EGFR amplification was associated with lymph node metastasis (*P* = 0.028), but not correlated with DFS and OS until 20 months.

**Conclusions:**

EGFR IHC overexpression evaluated by scoring system 1 might be suitable to be used in predicting patients survival in ESCC. EGFR gene amplification showed delayed prognostic information after 20 months.

**Electronic supplementary material:**

The online version of this article (doi:10.1186/s12885-015-1393-8) contains supplementary material, which is available to authorized users.

## Background

Esophageal carcinoma is one of the most common malignancies in China, and squamous cell carcinoma is the main histological type [1, 2]. It generally has a poor prognosis because it is usually in an advanced stage at the time of diagnosis. Despite the progress in chemotherapeutic, radiotherapeutic and surgical treatment, the five-year survival rate is still less than 20 % [3-6]. In recent years, molecular targeted therapy has become an important treatment [7-10]. With the aim of increasing the clinical benefit–risk ratio of anticancer treatments, consideration is increasingly given to the identification of predictive tumour biomarkers.

One potential group of useful protein biomarkers is the epidermal growth factor receptor (EGFR) family of receptors. This family contains four members, EGFR, ErbB2/human epidermal growth factor receptor-2 (HER2), ErbB3/HER3, and ErbB4/HER4, that act as receptor tyrosine kinases and have a well-defined function in cell signaling, controlling cell proliferation and differentiation. Esophageal cancers frequently show EGFR or HER2 gene amplification and overexpression [11, 12]. And esophageal squamous cell carcinomas (ESCCs) predominantly show alterations of EGFR, whereas esophageal (Barrett’s) adenocarcinomas (EACs) frequently show HER2 gene amplification and protein overexpression. In view of the prominent role in cancer cell biology and (over-)expression in substantial numbers of ESCC, EGFR represents valuable therapeutic target. Defining EGFR molecular characteristics relevant to patient prognosis is an important step toward deciding treatment.

At present, the literatures about EGFR expression in ESCC contain conflicting data on the relationship between overexpression and survival [13-15]. This variability may be due to heterogeneity of study populations or lack of a standardized assay for determining EGFR status. Here, we collected a cohort of Chinese patients with ESCC, and evaluated their protein expression using 6 representative scoring systems. To the best of our knowledge and available literature data, so far such comparisons of different EGFR-IHC scoring systems in ESCC patients are sparse.

EGFR gene copy number variation may be more reliable than protein expression in predicting prognosis. However, reports on the influence of EGFR gene variation in ESCC patients have been equivocal [13, 16-18]. In general, the relationships between tumor EGFR gene variation and protein expression have not been clearly defined, and the prognostic value of these tumor characteristics has not been well evaluated for ESCC.

Therefore, the aims of this study are to compare the six different scoring systems for EGFR expression, to explore the cut off value in assessing EGFR gene variation, and to investigate their prognostic significance in ESCC.

## Methods

### Patients and specimens

A total of 96 ESCC samples were treated in the Department of Thorax Surgery, Zhongshan Hospital during March to October 2010. All patients had not received chemotherapy or radiotherapy prior to surgical resection. Prior written informed consent was obtained from all patients. The present study has been carried out in accordance with the Declaration of Helsinki, and was approved by Human Research Ethics Committee of Zhongshan hospital, Fudan University.

Sections were stained with hematoxylin and eosin and reviewed by two pathologists to confirm the ESCC diagnosis. The following patient characteristics were collected: gender, age, tumor site (upper, middle, and lower region of esophagus), histological grade, coagulative necrosis, nerve and vascular infiltration, mitotic index (numbers recorded as ≤20 per 10 high power fields [HPF], 20-50/10HPF, or ≥ 50/10HPF), lymph node metastasis, and stage, as previously reported [19].

### Tissue microarrays

The tissue microarray (TMA) was constructed as previously described [20]. Briefly, the region of interest (2 mm wide and 6 mm long) was extracted and then vertically planted into the recipient block one by one according to the corresponding location indicated by letters and numbers. The planting surface was aggregated on the aggregation instrument.

### Immunohistochemistry

The TMA recipient block was sectioned on a routine microtome machine. The IHC assay using EGFR rabbit monoclonal antibody (EGFR.25, Leica Biosystems Newcastle Ltd, Newcastle, UK) was performed with the Ventana iView DAB Detection Kit on a BenchMark XT automated staining system (Ventana Medical Systems, Tucson, AZ). Normal IgG from the same species of primary antibody diluted to match the concentration of the primary antibody was used as the negative control. For EGFR negative cases, the experiment was repeated on the whole section in order to exclude heterogeneity.

EGFR expression was evaluated according to published scoring system, summarized as follows:1) The percentage of positive tumor cells (0 % to 100 %) was multiplied by the staining intensity (SI) (1, negative or trace; 2, weak; 3, moderate; 4, intense). Scores 0 to 200, 201 to 300, and 301 to 400 were respectively classified as having negative or low, intermediate, and high levels of expression [21]. 2) 0, negative, no discernible staining or background type staining; 1+, definite cytoplasmic staining and/or equivocal discontinuous membrane staining; 2+, unequivocal membrane staining with moderate intensity; 3+, strong and complete plasma membrane staining. Samples exhibiting 2+ or 3+ were classified as overexpression [13]. 3) a = 0 % (score 0); 1–20 % (score 1); 21–40 % (score 2); 41–60 % (score 3); 61– 80 % (score 4); or 81–100 % (score 5). i = absent (score 0); faint (score 1); moderate (score 2); or strong (score 3). A final score was calculated by multiplying i by a, using the score of 8 as the cutoff [22]. 4) 1 × (percentage of cells staining weakly [1 +]) + 2 × (percentage of cells staining moderately [2 +]) + 3 × (percentage of cells staining strongly [3 +]). Score of 200 is a cutoff [23]. 5) SI was classified as 0 (negative), 1 (weak), 2 (moderate), and 3 (strong). An area of SI was defined as 0 if <10 %, 1 if 10 %–25 %, 2 if 26 %–50 %, 3 if 51 %–75 %, and 4 if >75%. Immunostaining intensity was divided into 0 negative (−), 1–3 weakly positive (+), 4–5 moderately positive (2+), and 6–7 strong positive (3+); EGFR overexpression was defined as positive staining of tumor cells reaching 2+ or 3 + [24]. 6) loss of expression: SI = 0; weak expression: SI = 1 in < 70 % or SI = 2 in < 30 % of cells in a tumor spot, moderate expression: SI = 1 in > 70 % or SI =2 in > 30 % of cells in a tumor spot and strong expression: SI = 2 in > 70 % or SI = 3 in > 30 % of cells in a tumor spot [25].

### Fluorescence in situ hybridization

TMA sections were dewaxed and dehydrated. Dual color EGFR FISH was performed with the Spectrum Orange locus-specific identifier EGFR probe (Vysis, Abbott Molecular Inc, Des plaines, USA) specific for the EGFR locus (7p12) and the Spectrum Green CEP7 chromosome 7 centromeric probe (7p11.1 to q11.1; Vysis). The specific steps were similar to HER2-FISH procedure, reported previously [26].

EGFR signals were counted from at least 100 cancer cell nuclei, and were divided into six types: 1) disomy was an EGFR to CEP7 ratio ≤2 copies in >90 % of cells; 2) low trisomy was ≤2 copies in ≥40 % of cells, 3 copies in 10 %–40 % of the cells, ≥4 copies in <10 % of cells; 3) high trisomy was ≤2 copies in ≥40 % of cells, 3 copies in >40 % of cells, ≥4 copies in <10 % of cells; 4) low polysomy was ≥4 copies in 10 %–40 % of cells; 5) high polysomy was ≥4 copies in >40 % of cells; 6) gene amplification was defined by the presence of tight EGFR gene clusters, or a ratio of EGFR gene to chromosome7 ≥ 2, or ≥15 copies of EGFR per cell in ≥10 % of tumor cells. EGFR FISH-positive was defined as EGFR high polysomy or gene amplification [27].

### Follow-up information

Follow-up information for the 96 patients after surgery and treatment was provided by the referring clinicians, or else obtained directly from patients and their family members as standard procedure. The date of last follow up was May 16, 2014. Disease-free survival (DFS) and overall survival (OS) were measured from the time of surgery to the time of first recurrence (or most recent follow-up) or death.

### Statistical analysis

A χ2 test was used for univariate analysis, the agreement of different scoring systems was measured by the index Kappa and the statistical differences were analyzed by the McNemar test. Kaplan-Meier analysis was used to calculate DFS and OS. Log-rank test of survival analysis was used to compare DFS and OS as functions of variables and to identify significant differences. *P* < 0.05 were recorded as significant.

## Results

### Characterization of ESCC patients

The clinicopathological features of the 96 ESCC patients are summarized previously [28]. The majority of the patients were males (83.3 %). The median age of patients was 62 years. By anatomic site, 1 was located in the upper esophagus, 33 in the middle and 62 in the lower area. Most of the tumor differentiation was grade II (63.5 %), 36.5 % was grade III and none was grade 1. Five tumors had invaded to the submucosa, 24 to the muscularis propria and 67 to the adventitia. Fifty-three tumors were associated with nerve or vascular infiltration and 44 with lymph node metastases.

### EGFR IHC analysis

Among the 96 ESCC cases analyzed, the EGFR IHC staining results are evaluated using six scoring systems (Additional file 1: Table S1). The scoring system 1 and 6 has low, intermediate and high level of EGFR expression, while 2, 3, 4 and 5 only has low and high level (Table 1).

**Table 1:** Comparison of EGFR-IHC results of 6 scoring systems

Pairs	Levels	Number	*P*	Kappa
System 1 vs. System 6	(L/M/H) vs. (L/M/H)	(49/39/8) vs. (14/33/49)	0.001	0.037
System 2 vs. System 3	(L/H) vs. (L/H)	(42/54) vs. (48/48)	0.031	0.875
System 2 vs. System 4	(L/H) vs. (L/H)	(42/54) vs. (88/8)	0.001	0.132
System 2 vs. System 5	(L/H) vs. (L/H)	(42/54) vs. (12/84)	0.001	0.31
System 3 vs. System 4	(L/H) vs. (L/H)	(48/48) vs. (88/8)	0.001	0.167
System 3 vs. System 5	(L/H) vs. (L/H)	(48/48) vs. (12/84)	0.001	0.25
System 4 vs. System 5	(L/H) vs. (L/H)	(88/8) vs. (12/84)	0.001	0.026

The McNemar Test, *P* < 0.05 was considered statistically significant difference

Kappa > =0.75 was regarded as better concordance, Kappa < 0.4 indicated a poor concordance

System 1–6, EGFR-IHC scoring system 1 to 6

L, low level of EGFR expression; M, intermediate level; H, high level

**Fig. 1:**
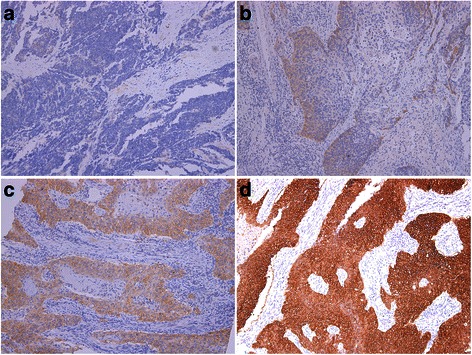
Examples of different immunohistochemical EGFR expression in ESCC according to system 1: **a** = negative control, **b** = low, **c** = intermediate, **d** = high level of EGFR expression

According to scoring system 1 to 6, overexpression of EGFR were observed in 8 (8.3 %), 64 (66.7 %), 48 (50 %), 8 (8.3 %), 84 (87.5 %), and 49 (51.0 %) cases, respectively (Table 1). Within the 6 scoring systems, 17 cases had the same level of EGFR expression (7 in high level, 10 in low level, and none in intermediate level) (Additional file 1: Table S1). Fig. 1a showed the negative control, 1b, 1c, and 1d were low, intermediate and high level of EGFR expression according to system 1.

There were significant difference among the six IHC score results (*P* < 0.001) (Table 1). The scoring system 2 and 3 are highly in agreement with each other (k = 0.87). No comparison could be conducted between 1 and 4.

### EGFR gene copy variation

**Fig. 2:**
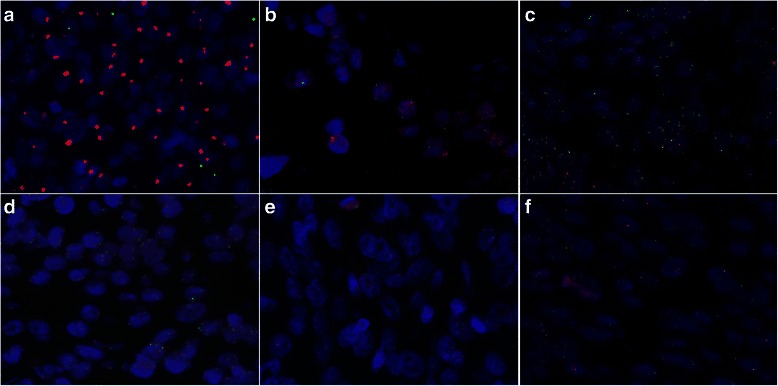
The representative EGFR (red) and chromosome 7 (green) FISH for tumors with EGFR gene amplification (**a** and **b**, n = 7/96), High polysomy (**c**, n = 22/96), Low polysomy ( **d**, n = 39/96), high trisomy (**e**, n = 6/96) and disomy (**f**, n = 3/96)

The average gene copy number per cell and the EGFR-to-chromosome 7 ratio for the major FISH patterns are listed in Additional file 2: Table S2. EGFR FISH-positive was seen in 29 (30.2 %) cases. The EGFR genes were amplified in 7 (7.3 %) cases (6 were clustered-type signals and 1 were multiple scattered signals) (Figs. 2a and b). High polysomy (≥4 copies in >40 % of cells) was present in the other 22 (22.9 %) cases, with the averages of EGFR and chromosome 7 signals per cell ranging between 3.11 and 5.10 and the gene-to-chromosome ratio ranging from 0.82 to 1.84 (Fig. 2c). Low polysomy was present in 39 (40.6 %) cases with the averages of gene and chromosome copies per cell ranging from 2.43 to 3.27 and the chromosome-to-gene ratio ranging from 0.92 to 1.14 (Fig. 2d), disomy in 3 (3.1 %) cases, with the averages per cell for the gene and chromosome 7 copies ranging from 2.02 to 2.17 and the ratio of gene-to-chromosome from 0.97 to 1.04 (Fig. 2f), low trisomy in 19 (19.8 %) cases with the averages per cell for the gene and chromosome 7 copies ranging from 1.95 to 2.49 and the ratio of gene-to-chromosome from 0.74 to 1.12, and high trisomy in 6 (6.3 %) cases with the averages per cell for the gene and chromosome 7 copies ranging from 2.56 to 2.91 and the ratio of gene-to-chromosome from 0.95 to 1.02 (Fig. 2e). These cases were categorized as a FISH-negative group.

### Correlation between EGFR protein expression and gene variation

EGFR expression and gene copy number are analyzed and showed in Table 2. EGFR gene amplification was associated with EGFR protein overexpression in scoring system 1, 2, 3, 4, and 6, EGFR-FISH positive only in scoring system 1, 2 and 4.

**Table 2:** Correlation between EGFR protein expression and gene variation

	EGFR-FISH result					
EGFR-IHC	FISH positive			Amplification		
	No	Yes	*P*	No	Yes	*P*
System 1			0.034			0.001
L	39	10		9	0	
M	25	14		37	2	
H	3	5		3	5	
System 2			0.036			0.015
L	34	8		42	0	
H	31	21		47	7	
System 3			0.120			0.006
L	37	11		48	0	
H	30	18		41	7	
System 4			0.038			0.001
L	64	24		86	2	
H	3	5		3	5	
System 5			0.674			0.299
L	9	3		12	0	
H	58	26		77	7	
System 6			0.175			0.027
L	11	3		14	0	
M	26	7		33	0	
H	30	19		42	7	
Total	67	29		89	7	

System 1–6, EGFR-IHC scoring system 1–6

L, low level of EGFR expression; M, intermediate level; H, high level

FISH positive, EGFR gene amplification or high polysomy

On the basis of scoring system 1, 3 and 4, patients with trisomy and polysomy showed low mean IHC scores (206 and 197, 6.6 and 6.5, 94.6 and 100 respectively), whereas the mean IHC score increased when FISH abnormalities became more severe. The mean score was 348.6, 13.7 and 254.3 for patients with gene amplification (Additional file 3: Table S3).

### Prognostic implication of EGFR protein expression levels and gene variation

EGFR protein overexpression and gene amplification were statistically evaluated for correlation with established clinicopathological factors (Table 3). EGFR overexpression according to scoring system 1 and 4 was significantly correlated with the vascular invasion, lymph node metastasis (*P* < 0.05, Table 3). No such correction was observed using other scoring systems. EGFR amplification was associated with the lymph node metastasis (*P* = 0.028), while high polysomy wasn’t associated with this factor (*P* = 0.227).

**Table 3:** Relationship of status of EGFR in ESCC with the clinicopathological parameters

		EGFR-IHC result							EGFR-FISH result					
		System 1				System 4			FISH Positive			Amplification		
	N	L	M	H	*P*	L	H	*P*	No	Yes	*P*	No	Yes	*P*
Gender														
Male	80	42	32	6	0.724	74	6	0.509	52	28	0.022	74	6	0.861
Female	16	7	7	2		14	2		15	1		15	1	
Age														
<60	34	24	8	2	0.017	22	12	0.422	22	12	0.422	32	2	0.694
>60	62	25	31	6		45	17		45	17		57	5	
Tumor site														
Upper	1	0	1	0	0.181	1	0	0.607	1	0	0.313	1	0	0.413
Middle	33	12	17	4		29	4		20	13		29	4	
Lower	62	37	21	4		58	4		46	16		59	3	
T-stage														
T1	5	2	3	0	0.889	5	0	0.499	3	2	0.552	5	0	0.602
T2	24	13	9	2		23	1		15	9		23	1	
T3	67	34	27	6		60	7		49	18		61	6	
Vaso invasion														
No	77	34	35	8	0.020	69	8	0.142	54	23	0.884	70	7	0.172
Yes	19	15	4	0		19	0		13	6		19	0	
Nerve invasion														
No	62	34	24	4	0.497	59	3	0.094	44	18	0.735	59	3	0.212
Yes	34	15	15	4		29	5		23	11		30	4	
LN metastases														
No	52	29	22	1	0.046	51	1	0.013	39	13	0.227	51	1	0.028
Yes	44	20	17	7		37	7		28	16		38	6	
Necrosis														
No	38	21	16	1	0.258	36	2	0.378	27	11	0.828	37	1	0.155
Yes	58	28	23	7		52	6		40	18		52	6	
Mitoses (/10HPF)														
≤20	29	12	15	2	0.645	27	2	0.307	18	11	0.06	27	2	0.768
20~50	37	20	13	4		32	5		31	6		35	2	
≥50	30	17	11	2		29	1		18	12		27	3	
Tumour differentiation														
G2	61	33	26	2	0.061	43	18	0.844	43	18	0.844	57	4	0.715
G3	35	16	13	6		24	11		24	11		32	3	

System 1and System 4, EGFR-IHC scoring system 1 and 4

L, low level of EGFR expression; M, intermediate level; H, high level

FISH positive , EGFR gene amplification or high polysomy

**Fig. 3:**
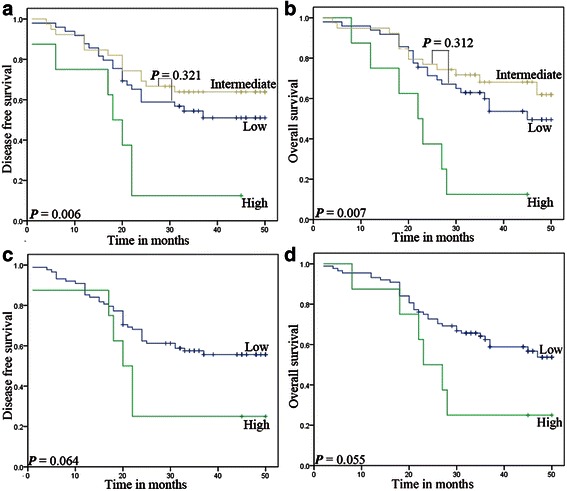
Association between EGFR overexpression and survival in ESCC. Protein overexpression, on the basis of scoring system 1, had poorer DFS (**a**, *P* = 0.006) and OS (**b**, *P* =0.007), with system 2 (**c** and **d**) no prognostic value

**Fig. 4:**
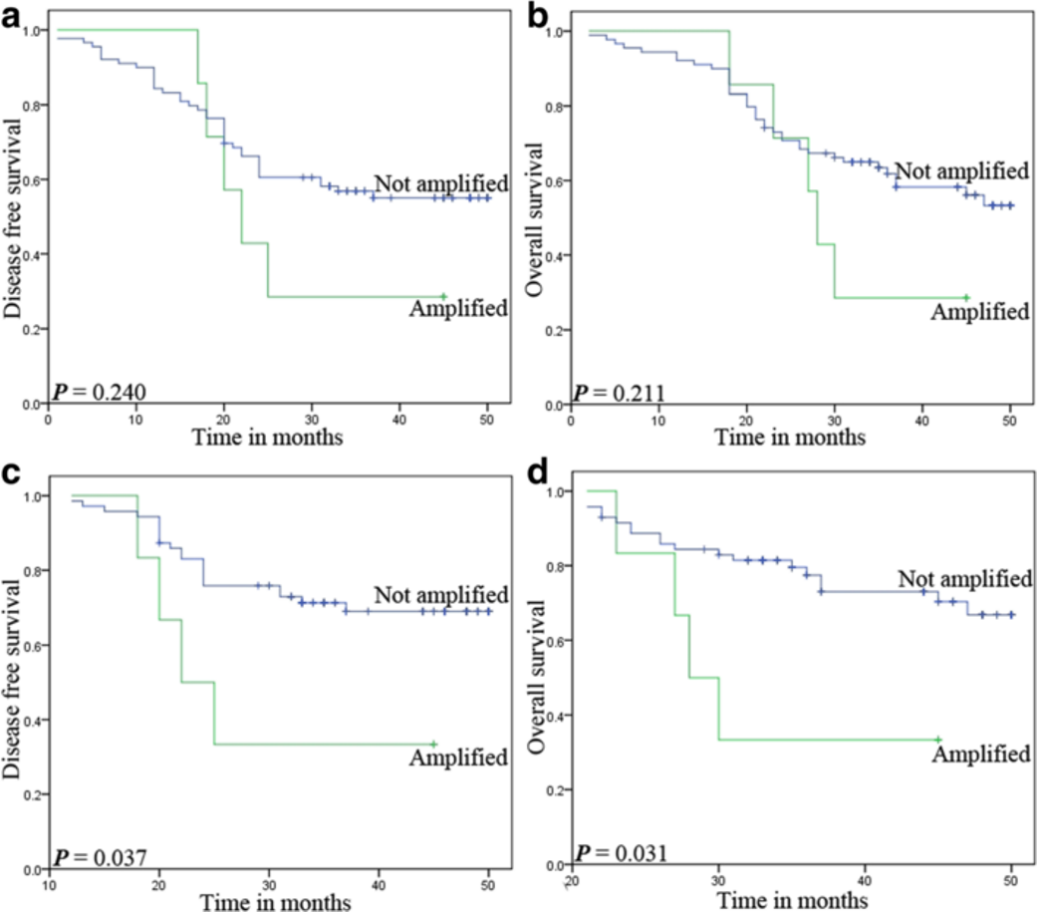
Association between EGFR gene variation and survival in ESCC. The gene amplification (**a** and **b**) was not significantly associated with DFS or OS (*P* = 0.240 or 0.211). However, gene amplification (**c** and **d**) did represent delayed prognostic information (*P* = 0.037 and 0.031)

EGFR overexpression and gene amplification were evaluated for their potential prognostic significance. The Kaplan-Meier survival curves for patients in the different scoring systems of EGFR expression or gene numbers are shown in Figs. 3 and 4. Protein overexpression, on the basis of scoring system 1, had poorer DFS (*P* = 0.006) (Fig. 3a) and OS (*P* = 0.004) (Fig. 3b). However, other systems had no prognostic value whether in DFS or in OS (Fig. 3c and d). And gene amplification did not represent a statistically significant adverse prognosis until 20 months (Fig. 4). No significant difference in survival rates with respect to high polysomy was observed.

## Discussion

The epithelial growth factor receptor (EGFR) is a 170-kDa transmembrane glycoprotein and a tyrosine kinase receptor expressed in various human tissues, especially in cells of epithelial origin, which plays important roles in modulating cell proliferation, survival, migration, and differentiation [29]. EGFR alterations in cancer can be divided mostly in two categories: mutations in exons 18–21 mainly identified in Asia lung adenocarcinoma [30, 31], and gene and protein overexpression [32]. It’s known to us, ESCC predominantly show EGFR gene copy number alterations and protein overexpression [11, 13, 24, 33], with little EGFR mutation [16, 26, 34]. EGFR gene variation and protein overexpression might be the candidate for predictive biomarker in ESCC. There have been several IHC studies examining EGFR protein expression in ESCC, the expression rate ranged from 4 % to 86 % [13, 24, 33]. We found the most important discrepancies might be due to the selected threshold for positivity, which may induce conflicting results in different laboratories and authors. Therefore, we selected six different scoring systems presented in literature to focus upon the same ESCC samples with EGFR antibody.

### Evaluation of EGFR expression by six scoring systems in ESCC

Firstly, the overexpression of EGFR were observed in 8 (8.3 %), 64 (66.7), 48 (50 %), 8 (8.3 %), 84 (87.5 %), and 49 (51.0 %) cases according to system 1 to 6, with ranging from 8.3 % to 87.5 %. These results were in agreement with our speculation that EGFR expression was obviously influenced by the selected threshold. Secondly, the correlation of EGFR expression with clinical features and prognosis were evaluated by scoring system 1 to 6, 1 and 4 related to lymph node metastasis, however, only 1 showed a statistically significant prognosis with DFS (0.006) and OS (0.007). Therefore, scoring system 1 for EGFR expression seems to be more valuable for predicting tumor aggressiveness and prognosis.

### Evaluation of EGFR gene variation in ESCC

Firstly, EGFR gene status disclosed by our FISH included disomy, low trisomy, high trisomy, low polysomy, high polysomy and gene amplification, which was consistent with previous reports [24]. Secondly, EGFR gene amplification was associated with EGFR expression evaluated by scoring system 1, 2, 3, 4, 6 except 5, whereas EGFR-FISH positive was only associated with scoring system 1, 2 and 4. Thirdly, EGFR-FISH positive had no relationship with clinical features and prognosis; however, EGFR amplification was associated with lymph node metastasis, and patients with EGFR amplification had poorer prognosis whether in DFS or OS after 20 months survival. Therefore, EGFR amplification, not EGFR-FISH positive or high polysomy, seems to be a suitable cut off value in clinical practice.

## Conclusion

This study firstly compared six scoring system evaluation for EGFR IHC overexpression used in ESCC, and found scoring system 1 might be suitable to be adopted in clinical practice since the value in predicting patients’ survival. EGFR gene amplification was associated with protein overexpression in ESCC, and indicated poorer prognosis after 20 months survival.

## Additional files

Additional file 1: Table S1. EGFR immunohistochemical staining results evaluated using six scoring systems.
Additional file 2: Table S2. EGFR-FISH results of all cases.
Additional file 3: Table S3. EGFR IHC scores and gene variation.

## Abbreviations

ESCC: Esophageal squamous cell carcinoma
EAC: Esophageal adenocarcinoma
EGFR: Epithelial growth factor receptor
HER2: Human epidermal growth factor receptor-2
IHC: Immunohistochemistry
FISH: Fluorescent in situ hybridization
TMA: Tissue microarray
SI: Staining intensity
DFS: Disease-free survival
OS: Overall survival
